# A structured dataset of the federalist society’s public engagements

**DOI:** 10.12688/f1000research.161735.1

**Published:** 2025-02-14

**Authors:** Chad M. Topaz

**Affiliations:** 1 Institute for the Quantitative Study of Inclusion, Diversity, and Equity, Williamstown, MA, 01267, USA; 2 Williams College, Williamstown, Massachusetts, 01267, USA; 3 University of Colorado, Boulder, Colorado, 80309, USA

**Keywords:** law, courts, judges, judicial appointments, organizational dynamics

## Abstract

**Background:**

The Federalist Society, a leading conservative legal organization, has played a significant role in shaping the American judiciary for decades. Despite its influence, comprehensive empirical data on the organization remains scarce. We address this gap by systematically documenting 20,205 public events hosted by the Society from 1984 to 2024, with substantive coverage from 2007 onward.

**Methods:**

Following ethical best practices in data collection and ownership, we gathered event metadata—including titles, dates, locations, sponsors, topics, and speakers—via web scraping from the Federalist Society’s archives. The dataset is structured to facilitate analysis of event trends, co-speaking networks, and thematic shifts over time. To ensure data integrity, we performed validation, deduplication, and cleaning, resulting in a well-structured, high-quality dataset.

**Conclusions:**

This dataset provides an empirical foundation for examining the Federalist Society’s role in legal discourse. It is a resource for scholars in law, political science, sociology, gender studies, network science, and computational social science, enabling investigations into key themes of discussion, event participation patterns, demographic diversity, professional backgrounds, and more. Researchers can use it to study institutional influence, legal networks, and the evolution of conservative legal thought.

## Introduction

The Federalist Society for Law and Public Policy Studies, established in 1982, is a prominent conservative and libertarian legal organization with nearly 100,000 members dedicated to reforming the current legal order. ^1^ It has significantly influenced the American judiciary by advocating for originalism and textualism in constitutional interpretation. ^2^ The Society has played a pivotal role in the nomination and confirmation of federal judges, including Supreme Court justices. During President Donald Trump’s first tenure, its leadership collaborated closely with the administration to recommend judicial nominees, ^3^ resulting in the appointment of 234 federal judges with lifetime tenure under Article III of the U.S. Constitution, including three of the nine current Supreme Court justices. ^4^

Despite the Society’s well-documented influence on judicial appointments and legal discourse, there is a lack of comprehensive empirical data on it. ProPublica’s Nonprofit Explorer ^5^ provides some access to financial records through tax disclousures. Amanda Hollis-Brusky’s influential book *Ideas with Consequences* ^6^ analyzes the Society’s strategies and ideological reach. Still, most research has relied on qualitative analysis, interviews, or case studies rather than systematic, large-scale data. Empirical studies quantifying the Society’s influence remain scarce, and scholars have had to rely on fragmented sources when attempting to study its role in shaping the judiciary and legal thought. The absence of a structured, machine-readable dataset capturing the Society’s public engagements limits researchers’ ability to assess its operations and goals. Without a comprehensive dataset, it is difficult to rigorously analyze questions about the Society’s organizational reach, its ideological evolution, or its impact on professional pathways within the legal system.

To address this gap, we compiled and structured data on 20,205 public events hosted by the Federalist Society between 1984 and 2024, with substantive coverage beginning in 2007. The dataset contains detailed metadata, including event titles, dates, locations, sponsors, topics, and speakers, and is designed to facilitate quantitative and qualitative research on the Society’s role in shaping legal discourse. By making this data openly available, we aim to provide scholars with a robust empirical foundation for analyzing patterns of legal engagement, speaker networks, and thematic evolution within the Federalist Society’s programming.

### Motivation for creating the dataset

Our dataset provides a foundation for numerous lines of inquiry into the Federalist Society’s structure, influence, and evolution. Below, we outline seven areas of research that could be of interest to legal scholars, political scientists, network scientists, organizational theorists, gender scholars, sociologists, and computational social scientists.

First, researchers could examine how the Federalist Society’s presence has changed over time and across regions. Analyzing the geographic distribution of events could reveal areas of strong or limited activity, while tracking the number of unique speakers per year may indicate whether the network has expanded or remained insular. Shifts in event types and frequency—such as transitions from lectures to panel discussions or from in-person gatherings to virtual forums—may reflect broader trends in legal discourse and engagement strategies.

Second, mapping relationships among speakers could illuminate the structure of the co-speaking network. Identifying frequently recurring figures, as well as speakers who bridge multiple topic areas, would provide insight into key influencers within the organization. Tracking individual trajectories over time may also reveal whether certain speakers later attain judicial or political appointments. Additionally, this dataset enables an examination of professional clustering within the speaker network, revealing whether academics, practitioners, or judges tend to form distinct communities.

Third, event titles and descriptions offer a rich source of information for analyzing the substantive focus of the Federalist Society’s discussions. Using natural language processing, researchers could identify dominant themes, track how these themes evolve, and determine whether particular topics gain prominence following external events such as Supreme Court rulings or shifts in political leadership. Regional variations in topic selection could reveal how different chapters tailor their programming. The dataset also allows for an examination of whether larger panels tend to focus on particularly contentious or consensus-driven topics.

Fourth, the dataset provides an opportunity to study gender representation within the Federalist Society’s speaker network. Many biographical entries include titles (Mr., Ms., Mrs., etc.) or pronouns, which can be ethically leveraged to infer gender representation. Since these biographies are either self-provided or reviewed by the speakers themselves, this method respects individual identity. Researchers could analyze trends in gender balance over time, assess whether women are more or less likely to be repeat speakers, and evaluate how gender diversity varies across different topic areas. Co-speaking relationships could also be examined to determine whether speakers tend to appear alongside others of the same gender.

Fifth, biographical information about speakers’ affiliations makes it possible to assess the balance between academic, judicial, and practitioner speakers. A historical view of how these affiliations shift could reveal whether the Society has become more or less dominated by certain professional backgrounds. Furthermore, tracking career movements—such as transitions from academia to the judiciary—could shed light on the role of Federalist Society events in shaping legal careers.

Sixth, for those interested in statistical modeling, this dataset could help identify factors that predict speaker recurrence. Certain credentials or professional backgrounds may be associated with a higher likelihood of being invited back. The dataset also enables modeling of topic diffusion across the network, identifying patterns in the spread of legal and ideological discourse. The structure of event panels could be analyzed to determine what factors are associated with whether an event features a single speaker or a larger panel discussion.

Finally, this dataset supports investigations into how the Federalist Society’s programming responds to external political and legal developments. Certain topics may become more prevalent following major Supreme Court rulings, elections, or shifts in judicial appointments. Additionally, researchers could analyze whether certain speakers—particularly those who later become federal judges—appear more frequently alongside future nominees, offering insights into the Society’s role in shaping judicial careers.

## Methods

This dataset documents past public events organized by the Federalist Society. The dataset includes details about event titles, dates, locations, topics, sponsors, event descriptions, speaker names, and speaker biographies. We collected data programmatically through web scraping in order to ensure comprehensive coverage of all events listed on the Federalist Society’s website. We performed data collection on December 4, 2024. Our code is available in our Open Science Framework repository. ^7^ We carried out scraping and processing using open source tools: RStudio (version 2024.12.0+467) running R (version 4.4.2) with the pbmcapply, rvest, and tidyverse libraries. To optimize performance, we distributed data requests across 23 CPU cores on a Mac Studio M2 Ultra computer, leveraging parallel processing to expedite retrieval. Given the scale of the dataset—several tens of thousands of web pages—parallelization was essential to efficiently process web requests and prevent excessive runtime delays.

### Event data retrieval

To construct a dataset of public events hosted by the Federalist Society, we systematically collected event listings from their official website’s archive of past events. ^8^ On the day of data collection, we verified that the archive contained 2,044 pages of event listings, each (except for the last) containing 10 events. We iterated through each of these pages and extracted structured information by identifying relevant content elements. The collected fields included: event title; event date; event location; event topics; event sponsors; event type, *e.g.*, lecture, panel, or seminar; speaker names and biography links; and hyperlink to the event’s dedicated webpage. In total, we collected 20,433 event records.

### Event description retrieval

Each event listed on the Federalist Society’s website has a dedicated webpage containing additional textual descriptions that provide further context about the discussion topics and speakers. Using the 20,433 event URLs gathered in the previous stage, we accessed each event’s page and retrieved its description. If an event page contained multiple descriptive paragraphs, we concatenated them into a single text field. After extraction, we cleaned the data by (1) trimming leading and trailing whitespace, and (2) removing a boilerplate disclaimer about the Federalist Society’s neutrality, which appeared in some event descriptions. Finally, we merged the event descriptions into our main dataset.

### Speaker biography retrieval

To enhance event data with speaker details, we extracted biographical information for each individual listed as a speaker. In the previous stage, we collected speaker names and their associated biography URLs. We then accessed each speaker’s biography page and retrieved the full text of their profile. To avoid redundant processing, we identified 6,723 unique speakers among the 20,433 events and we processed each biography only once. If a biography page contained multiple text blocks, we concatenated them into a single entry. As in the previous stage, we performed additional data cleaning by trimming whitespace and removing a standard disclaimer stating that appearance on the site does not “imply any other endorsement or relationship between the person and the Federalist Society.” Finally, we assigned a unique speaker ID to each individual for consistent referencing across events and merged the speakers back into our main dataset.

### Data finalization

During data processing, we identified 385 events that appeared multiple times with the same title and date. A manual review confirmed that these were not scraping errors but genuine duplicate listings on the website. While these events shared identical titles and dates, their full records varied; some contained more abbreviated information than others. To ensure completeness, we consolidated these near-duplicate entries into a single record, retaining the most detailed version of the event description, address, and other key details.

In the final dataset, we flattened events with multiple speakers, meaning that each row represents one speaker at one event—an event-speaker pair. As a result, the dataset contains 30,898 rows, corresponding to 20,205 unique events and 6,723 unique speakers. The total number of events is lower than the 20,433 originally scraped because of merging near-duplicates.

The final dataset contains 16 fields:

• **event_id:** A unique identifier for the event. [integer]
• **speaker_id:** A unique identifier for the speaker. [integer]
• **event_title:** The title of the event. [text]
• **sponsors:** The sponsors of the event, such as Federalist Society student chapters, professional groups, and topical interest groups. [text]
• **topics:** Words or brief phrases describing the focus of the event, such as “Administrative Law & Regulation” or “Federal Courts.” These topics follow the Federalist Society’s own taxonomy. For events with multiple topics, entries are separated by a pipe “|”. [text]
• **event_types:** The medium for the event, such as “In-Person,” “Webinar,” or “Live Stream.” For events with multiple types, entries are separated by a pipe “|”. [text]
• **event_link:** The web address of the event’s page on the Federalist Society website. [text URL]
• **paragraph_content:** Unstructured information provided as part of the event description. [text]
• **speaker_name:** The name of the speaker. [text]
• **
speaker_bio_link:** The web address of the speaker’s biography page on the Federalist Society website. [text URL]
• **bio_content:** The speaker’s biography as posted online. [text]
• **year:** The year of the event. [integer]
• **month:** The month of the event. [integer]
• **day:** The day of the event. [integer]
• **address:** The location of the event, which could be a physical venue or an online reference. [text]
• **combined_flag:** Indicates whether the record resulted from merging two or more near-duplicate listings. [true/false]

## Dataset validation

Because this dataset was constructed through web scraping, traditional validation techniques—such as comparing against an external authoritative source—do not directly apply, as no comprehensive structured dataset of Federalist Society events and speakers exists for benchmarking. Instead, we ensured internal consistency, completeness, and accurate extraction of structured information by implementing duplicate resolution and manual verification of sampled records.

### Missing data and limitations

This dataset covers events from 1984 to 2024, but only 44 events (0.2%) date from 1984 through 2006. Since the Federalist Society could not have maintained an online event archive in the earliest years, these records must have been added retroactively. There is a sharp increase—from 9 events in 2006 to 357 in 2007—which likely reflects a shift to systematic online record-keeping. Alternatively, it could represent a sudden, significant expansion in the number of events the organization hosted. Further archival research would be needed to determine the precise cause. Regardless, researchers should treat 2007–2024 as the dataset’s effective coverage period.

Because web scraping captures only what is publicly available, any missing event descriptions, speaker biographies, or other metadata on the Federalist Society’s site at the time of collection remain missing in the dataset. For example, some events ( *e.g.*, annual conventions, holiday parties, and networking receptions) do not list individual speakers, and some speaker biography pages exist without any content beyond a standard disclaimer. Table 1 documents the extent of missing data and provides likely explanations.

**Table 1.** Summary of missing data across key fields in the dataset. The columns are: variable; number and percentage of missing records; number and percentage of unique events affected; and likely explanation. Gaps reflect structural patterns in event documentation rather than data collection artifacts. For example, networking events and large conventions often lack listed speakers, while some speaker biography pages exist without biographical content beyond a standard disclaimer.

Variable(s)	Missing records	Missing unique events	Likely explanation
all speaker info	2,512 (8.1%)	2,512 (12.4%)	Events without named speakers, such as networking events or multi-session conferences. Examples include “2011 National Lawyers Convention,” and “2019 Summer Social.”
bio_content only	5,588 (18.1%)	4,358 (21.6%)	Some speakers have a biography link but no biographical text, only a disclaimer stating that “appearance does not imply any other endorsement or relationship between the person and the Federalist Society.”
sponsors	733 (2.4%)	330 (1.6%)	Some events do not list sponsors, particularly broad-topic panels or private events.
topics	8,161 (26.4%)	6,075 (30.0%)	Some events do not include topical labels, especially informal gatherings.
event_type	3,233 (10.5%)	1,553 (7.7%)	Some events do not specify a format ( *e.g.*, lecture, panel, seminar).
paragraph_content	5,586 (18.1%)	4,069 (20.1%)	Some events lack descriptions beyond title and date.
address	7,157 (23.2%)	4,864 (24.1%)	Online events and some others lack detailed location information.

For speaker biographies, we further validated the missing cases by randomly sampling 60 speakers (approximately 1% of total) and verifying that in each case, the speaker’s webpage existed but contained no biographical text aside from a disclaimer stating that “appearance does not imply any other endorsement or relationship between the person and the Federalist Society.”

While missing data is often viewed as a technical limitation, in this case, it may reflect meaningful institutional patterns in how the Federalist Society documents its events. For instance:

• Certain types of events, such as holiday gatherings and networking receptions, rarely list speakers.
• The absence of speaker biographies could indicate variation in how the organization archives professional credentials or how individuals choose to share information rather than a data collection artifact.
• Gaps in event topics and sponsors may correspond to real differences across event types rather than accidental omission.

Future research could systematically analyze these patterns to assess how the Federalist Society’s event documentation practices vary over time and across event categories.

### Validation steps

To ensure the accuracy of the dataset, we performed the following checks:

• **URL Verification:** We confirmed that every link we followed during scraping—event listing pages, individual event pages, and speaker biography pages—existed at the time of data collection. To restate, all URLs led to pages that actually existed.
• **Manual Review:** We manually checked a random subset of 300 records (approximately 1% of total) to verify that extracted text fields, such as event titles, descriptions, and speaker biographies, matched the content displayed on the website. We found no discrepancies.
• **Duplicate Handling:** As described earlier, we identified and consolidated 385 near-duplicate event listings, preserving the most complete version of each event.

In summary, this dataset represents the complete publicly available event record as of December 4, 2024, though it substantively covers the period from 2007 to 2024. While missing data presents some limitations, it also provides insight into how the Federalist Society documents and categorizes its events. More broadly, this dataset enables empirical analysis of the organization’s event structure, speaker networks, and thematic focus, offering a foundation for studying its operations and influence.

## Ethical considerations

This study utilizes a dataset compiled from publicly available information sourced from the Federalist Society’s website. The dataset consists of historical records of speaking events, which were systematically extracted, structured, and processed for research purposes. Below, we address key ethical and legal considerations regarding data ownership, informed consent, and compliance with research integrity standards.

### Public availability and data collection

The event records included in this study were publicly accessible at the time of collection, requiring no authentication, login credentials, or restricted access. The information was posted by the hosting organization as part of its publicly available event listings. No private, sensitive, or confidential data were accessed or collected.

At the time of data collection, the Federalist Society’s robots.txt file permitted general web crawling but restricted automated access to certain types of URLs, including those containing query parameters related to speakers, locations, topics, and media. Our data collection adhered to these restrictions by scraping only publicly available event listing pages that were not disallowed. We made no attempts to bypass these limitations, and we structured automated requests to avoid undue server load.

### Data ownership and generation

The original information in this dataset was spread across thousands of web pages and did not previously exist in structured form. Under U.S. copyright law, factual data is not copyrightable. The Supreme Court’s decision in *Feist Publications, Inc. v. Rural Telephone Service Co.* ^9^ established that mere collections of facts lack the originality required for copyright protection. Applying this principle, the underlying event details in this dataset—such as event titles, dates, and speaker names—are considered public domain information. Moreover, no database rights exist under U.S. law that would grant exclusive control over a compilation of publicly available facts.

The dataset used in this study was created through systematic extraction, processing, and reorganization of public information, resulting in a structured resource designed for research purposes. This approach aligns with established academic practices in computational social science and legal research, where publicly available data is routinely collected and analyzed. The organization, cleaning, and structuring of the data constitute an original compilation, distinguishing it from a simple reproduction of raw records. Such methodologies are widely recognized in academic literature as valuable tools for research. Recent work proposes a comprehensive framework for web scraping in social science research, highlighting its role in expanding data access while addressing legal, ethical, and scientific considerations. ^10^ This work discusses how unstructured web data, when systematically collected and processed, can support rigorous empirical analysis—as long as researchers adhere to best practices in transparency, validity, and ethical responsibility.

### Informed consent and privacy considerations

This study does not involve human participants in a way that requires informed consent, as defined by the Declaration of Helsinki ^11^ and typical institutional research ethics guidelines. The dataset contains only publicly available event information, with no personally sensitive data or private records. No interaction with individuals took place, and no personally identifiable information beyond publicly listed event details was collected.

### Legal and ethical compliance

At the time of data collection, the Federalist Society website did not publish any terms of service that restricted the use of publicly available data for research purposes. The absence of contractual limitations means that no restrictions apply. Additionally:

• We made no attempt to bypass any technological barriers or access restricted content.
• The dataset contains no social media data, which would require additional ethical considerations.
• We present the dataset without any misrepresentation of the original information.

In summary, this research aligns with best practices in open data, transparency, and ethical scholarship.

## Data Availability

Open Science Framework: A Structured Dataset of the Federalist Society’s Public Engagements https://doi.org/10.17605/OSF.IO/QH672. ^7^ This project contains the following underlying code files and data:
•
**scrapeEventList.R** – script to gather basic information about events •
**scrapeParagraphText.R** – script to gather event description •
**getBios.R** – script to gather biographical information •
**combineFedsoc.R** – script to combine and finalize data •
**fedsoc.csv** – final data after gathering, structuring, processing, and cleaning Data are available under the terms of the Creative Commons Attribution 4.0 International license (CC-BY 4.0).
