# Supplementary material for: A structured dataset of the federalist society’s public engagements
Source: F1000Res. 2025 Jul 25;14:213. Originally published 2025 Feb 14. [Version 2] doi: 10.12688/f1000research.161735.2 (PMC12203041; doi:10.12688/f1000research.161735.2)
Supplement: Peer review history

# Reviewer report: Reviewer response for version 1

**DOI:** 10.5256/f1000research.177808.r373577

**Published:** 2025-06-26

**Authors:** Malcolm Langford

**Affiliations:** 1 University of Oslo, Oslo, Norway

This dataset provides an important insight into a powerful institution in American civic life, especially in the appointment of and influence on American judges. I would recommendation indexing and make only the following minor comments.

 1. While it is explained that missing data stems from "structural patters in event documentation rather than data collection artifacts", there seems to be qualitative and important differences between the missing categories. The table seems to cobble together missing data that results from construct validity decisions (e.g., "topics", which would be irrelevant for informal gatherings) and data content (e.g., bio_content_only).

 2. I am not convinced that most users can generate gendered categories for speakers. This usually requires more advanced computational skills and there is a rich literature and set of methods that concern how to address ambiguous and rare names. I think the author could create a gendered variable.

 3. The privacy considerations are US-centric, but the data may be used outside the United States. Storage and use of this dataset would require data privacy applications in many EU/EFTA jurisdictions as it contains personal data. Thus, the text should indicate that this assessment relates solely to US law.

 4. There should be a discussion of other relevant datasets to which this dataset could be linked, especially those concerning judgments and judges, and how that might be best done.

 5. If space permits, it could be helpful for readers to see some examples of applications of the different methods proposed under the seven motivations. E.g., one example with the scholarly reference.

Are sufficient details of methods and materials provided to allow replication by others?

Yes

Is the rationale for creating the dataset(s) clearly described?

Yes

Are the datasets clearly presented in a useable and accessible format?

Yes

Are the protocols appropriate and is the work technically sound?

Yes

Reviewer Expertise:

Comparative constitutional law; Empirical legal studies; Computational legal studies; Legal profession studies

I confirm that I have read this submission and believe that I have an appropriate level of expertise to confirm that it is of an acceptable scientific standard.

# Response

**Published:** 2025-07-21

**Authors:** Chad Topaz

**Affiliations:** Humanities, Williams College, Williamstown, Massachusetts, USA

Thank you very much for your thoughtful review and supportive recommendation. I appreciate your engagement with the dataset and your helpful suggestions for improving its clarity, accessibility, and contextualization. Below, I respond to each of your comments.

**Differentiating types of missing data. **Thank you for highlighting the qualitative differences among the missing fields. I have revised the *Missing Data and Limitations* section to clarify the distinction between construct-driven omissions (e.g., event topics not listed for informal gatherings) and content-absent fields (e.g., speaker biographies that exist only as disclaimers). This clarification helps contextualize missingness as a function of institutional documentation practices rather than simple data loss.

**Gender inference. **I appreciate your suggestion to include a gender variable to support gender-based analyses. However, I have chosen not to infer or assign gender in the dataset. Inferring gender from names, titles, or pronouns can result in misclassification, particularly for nonbinary individuals or others whose identities may not be accurately captured by such heuristics. Because these decisions carry ethical weight and the potential for harm, I believe they are best made by individual researchers whose specific analytic goals require them. To support such work, the dataset includes full speaker biographies and clear documentation of their structure and missingness.

**Privacy considerations outside the United States. **Thank you for raising this important point. I have added a clarifying sentence to the *Ethical Considerations* section noting that while the dataset complies with data use and privacy standards in the United States, researchers in other jurisdictions—particularly in the European Union and the European Free Trade Association, where the General Data Protection Regulation applies—should ensure that their use and storage of the dataset complies with local data protection laws.

**Linkages to other datasets. **I appreciate your recommendation to discuss possible linkages with other relevant datasets. I have added a bulleted list at the end of the *Potential Research Applications* section describing five publicly available resources that researchers may wish to link to this dataset. These include the Federal Judicial Center’s biographical directory, the Supreme Court Database, the Caselaw Access Project, the Oyez Project, and OpenSecrets. Each entry includes a brief description and web address to facilitate access.

**Examples of analytic methods. **Thank you for the suggestion to provide concrete examples of analytic approaches. In keeping with the scope of this paper as a dataset description, I have chosen not to include specific analyses or methods beyond the use cases already described. My aim is to provide a flexible foundation that enables a wide range of research questions without predefining the analytic approaches researchers should take.

 Thank you again for your valuable and thoughtful feedback, which helped strengthen the clarity, usability, and contextual framing of the dataset.

# Reviewer report: Reviewer response for version 1

**DOI:** 10.5256/f1000research.177808.r366981

**Published:** 2025-03-05

**Authors:** Jonathan Kropko

**Affiliations:** 1 University of Virginia, Charlottesville, USA

This dataset is a comprehensive collection of events hosted by the Federalist Society from 1984 to 2024 (with better coverage from 2007 onward), along with the speakers, the event titles/types and topics, and the published biographical information about the speaker. It is collected via webscraping of the Federalist Society's website using R (and the pbmcapply, rvest, and tidyverse packages). It was collected in accordance with the restrictions outlined in the website's robots.txt file. The dataset is the first structured dataset of Federalist Society events and speakers.

 This work is a significant contribution to quantitative scholarship on the American legal system as it intersects with politics. It may be argued that because the dataset focuses on only one organization that the scope of the data is too narrow. However, the Federalist Society has an outsized impact on American jurisprudence and is responsible for advancing affiliated individuals to three seats on the current Supreme Court as well as many federal judgeships. The Federalist Society is more than worthy of study in its own right and this dataset will facilitate that study.

 I recommend that the work may be indexed as is. However, if the authors will be submitting revisions, there are three areas that may be addressed to strengthen the contribution.

 First, while the authors do state that the speaker bio_content often contains information about the speaker's gender, they leave it to the data user to extract this information from pronouns and salutations. I imagine that while some of the users of this dataset are capable of extracting this information properly, many social scientists and legal scholars are not well trained in working with text data and may be held back by this requirement. The authors are highly proficient in data wrangling, and it may be an excellent additional benefit to users if the authors could also construct a speaker_gender column.

 Second, the event topics are currently stored in a list format where each of the event's topics are separated by a pipe | symbol. Some researchers however, especially those who will be using the data to do predictive modeling, will likely be adapting the data into a relational database. According to the rules of first normal form, the topics as currently stored are nonatomic and will need to be moved to another data table that has one topic per row along with the event ID. This table would also make it much easier for users to create bar charts of topic frequency, for example. Instead of forcing users to do this, the authors can create this table and make it separately available.

 Third, the data contains a unique speaker_ID. However, because many of the speakers are or go on to be federal judges, users may want to link this dataset with a separate dataset that tracks judges, such as https://www.fjc.gov/history/judges/biographical-directory-article-iii-federal-judges-export. A user who wants to merge the data with the FJC data would currently have to do so on the speaker/judge's name, which is a difficult and error prone method. If the authors can include additional columns for external judge IDs, such as the nid and jid columns in https://www.fjc.gov/sites/default/files/history/judges.xlsx, for example, then it would facilitate a great many more kinds of analyses from the data.

Are sufficient details of methods and materials provided to allow replication by others?

Yes

Is the rationale for creating the dataset(s) clearly described?

Yes

Are the datasets clearly presented in a useable and accessible format?

Yes

Are the protocols appropriate and is the work technically sound?

Yes

Reviewer Expertise:

data science, data engineering, political science

I confirm that I have read this submission and believe that I have an appropriate level of expertise to confirm that it is of an acceptable scientific standard.

# Response

**Published:** 2025-07-16

**Authors:** Chad Topaz

**Affiliations:** Humanities, Williams College, Williamstown, Massachusetts, USA

Thank you very much for your thoughtful and constructive feedback. I’m especially grateful for the care with which you engaged the structure and potential uses of the dataset. Below, I respond to each of your suggestions.

**Gender inference from speaker biographies.** I appreciate your suggestion to include a speaker gender column to support gender-related analysis. However, I have chosen not to include such a column in the dataset. Inferring gender from names, titles, or pronouns—while technically feasible—can lead to misclassification, particularly for nonbinary individuals or others whose identities may not be accurately captured by such heuristics. Because these decisions involve ethical considerations and the potential for harm, I believe they are best made by individual researchers whose analytic goals require them.

**Normalization of the event topics field.** Thank you for this suggestion. I agree that a normalized event–topic table would be useful for users working with relational databases or conducting topic-level analysis. In response, I’ve created a supplemental file that contains one row per event–topic pair. This file makes it easier to perform frequency counts, joins, and other analyses while preserving the original flat file for broader accessibility. I’ve described this supplemental table in the manuscript and included it in the publicly available project repository.

**Linking to external judge identifiers.** I appreciate the suggestion to include external judge IDs (e.g., nid, jid) to facilitate linkages with the Federal Judicial Center’s biographical directory. However, I’ve chosen not to add those identifiers. Doing so would require matching speaker names and biographical details across datasets. While the result would be useful, the process is susceptible to errors. Because the dataset is designed to reflect only what is available from the Federalist Society’s public archive, I believe it’s important to preserve that boundary. Researchers interested in merging with external sources are welcome and encouraged to do so based on the provided speaker names and biographies.

 Thank you again for your constructive and valuable feedback, which helped improve the clarity and usability of the dataset.
